# miRNA: A Promising Therapeutic Target in Cancer

**DOI:** 10.3390/ijms231911502

**Published:** 2022-09-29

**Authors:** Amrutha Menon, Noraini Abd-Aziz, Kanwal Khalid, Chit Laa Poh, Rakesh Naidu

**Affiliations:** 1 School of Pharmacy, Monash University Malaysia, Jalan Lagoon Selatan, Bandar Sunway 47500, Selangor Darul Ehsan, Malaysia; 2 Centre for Virus and Vaccine Research, School of Medical and Life Sciences, Sunway University, Bandar Sunway 47500, Selangor Darul Ehsan, Malaysia; 3 Jeffrey Cheah School of Medicine and Health Sciences, Monash University Malaysia, Jalan Lagoon Selatan, Bandar Sunway 47500, Selangor Darul Ehsan, Malaysia

**Keywords:** miRNA, cancer, tumor suppressor, oncomiR, therapy, miRNA delivery

## Abstract

microRNAs are small non-coding RNAs that regulate several genes post-transcriptionally by complementarity pairing. Since discovery, they have been reported to be involved in a variety of biological functions and pathologies including cancer. In cancer, they can act as a tumor suppressor or oncomiR depending on the cell type. Studies have shown that miRNA-based therapy, either by inhibiting an oncomiR or by inducing a tumor suppressor, is effective in cancer treatment. This review focusses on the role of miRNA in cancer, therapeutic approaches with miRNAs and how they can be effectively delivered into a system. We have also summarized the patents and clinical trials in progress for miRNA therapy.

## 1. Introduction

Like transcription factors, non-coding RNAs can also modulate the expression of genes. MicroRNAs are small non-coding RNAs that, since their discovery in 1993, have shown to be capable of modulating the activity of genes [1,2]. They bind to miRNA transcripts by Watson–Crick pairing and thus inhibit gene expression [3]. They are involved in a myriad of functions which include development, proliferation, differentiation, apoptosis, stress resistance, fat metabolism, survival and the tumorigenesis and metastasis of cells [4]. The effect of miRNA gene expression that often happens post-transcriptionally is termed as gene silencing and is established mainly by mRNA cleavage, translational repression, or DNA methylation [5,6]. RNase-like enzymes facilitate miRNA-mediated transcript cleavage, which has a significant effect on the resulting mRNA expression [7]. Translational repression regulated by RNA helicases is very imperative for RNA degradation [8]. The association between DNA methylation and gene silencing was first studied in plants [1]. miRNAs were found to be regulating the methylation of DNA by targeting DNA methyltransferase enzymes [9]. Epigenetic modification of genes like this has now been identified as being associated with tumor development [10,11]. It was known earlier that miRNAs bind to their target gene and repress their function by binding only to the 3′ untranslated regions [12,13,14]. However, Jopling et al. illustrated that miR-122 in liver cells can bind to the 5′ non-coding region of hepatitis C viral RNA. Supporting this finding, Orom et al., have shown that miR-10a can bind to the 5′ untranslated region of its target mRNA and induce gene regulation, thus confirming that both 3′ and 5′ non-coding region can act as an miRNA binding site [15].

In 1993, Lee et al., discovered miRNA in *Caenorhabditis elegans,* which is transcribed from gene *lin-4* and has complementarity to and hence regulates lin-14 protein expression [2]. Later in vivo studies proved that lin-4 microRNA targets lin-14, causes the termination of UNC-6 (netrin)-mediated axon guidance and regulates growth and development in *C. elegans* [4,16,17]. Later in 2000, Brenda et al., discovered another miRNA, let-*7*, in *C. elegans* itself that regulates proteins that are active in the developmental stage. This could lead to discoordination in stage-specific events during development [16,17].

Until recently, miRNAs were disregarded just as fragments of RNA, but the discovery of their aberrant expression in a wide array of pathological events and their involvement in carcinogenesis has recently attracted much attention. They were found to play important functions as oncomiRs by inhibiting several tumor-suppressing genes or as tumor suppressors by regulating oncogenes [18,19]. Interestingly, some miRNAs have been identified as having dual functional roles, wherein they act as a tumor suppressor in one type of cancer and as oncomiR in the other. For example, miR-10b acts as an oncomiR in GBM and is linked to poor overall survival. mRNA targets of miR-10b include p21, p16, BIM and TFAP2C. Silencing miR-10b was found to reduce the growth rate and increase the rate of apoptosis of glioma cells [20,21]. On the contrary, it has been identified that the same miRNA, miR-10b, acts as a tumor suppressor and is downregulated in cervical cancer, gastric cancer and small cell carcinoma [22,23,24].

## 2. miRNA Biogenesis

The biogenesis of miRNAs that are transcribed as independent units begins with the generation of primary-miRNA transcript (Pri-miRNA) by RNA Polymerase II or III [25]. Pri-miRNA consists of a large flexible terminal loop (≥10 bp) and a flanking single-stranded sequence of up to one kilobases with capping at the 5′ end and a polyadenylated tail at the 3′ end [26]. Later, pri-miRNA gets cleaved by RNaseIII Drosha to form 80–100-nucleotide-long intermediary precursor miRNA (pre-miRNA) [27,28]. To facilitate pri-miRNA cleavage, Drosha can form two complexes, one comprising RNA helicases p72 and p68 and the heterogeneous nuclear ribonucleoproteins (hnRNPs), the other called a microprocessor comprising Drosha and DiGeorge syndrome Critical Region 8 protein (DGCR8) [29,30]. DGCR8 facilitates the exact cleavage of pri-miRNA to remove 11bp from the hairpin stem [31]. Pre-miRNA is then transported from the nucleus into the cytoplasm in a GTP-dependent process by Exportin-5, which is a Ran-GTP-dependent dsRNA binding protein [32]. Exportin-5 also prevents pre-miRNAs from degradation [33]. Translocated pre-miRNA is then digested by Dicer and another RNase III to form a 22nt mature duplex miRNA (miRNA-miRNA*), which later unwinds into two separate strands (passenger strand (miRNA*) and guide strand (miRNA)) through an ATP-dependent process [34]. To increase the digestion activity and stability, Dicer also binds with other proteins such as the TAR RNA binding protein (TRBP and kinase R-activating protein) [35,36]. Later, in an ATP-independent process, the duplex unwinds, the passenger strand gets degraded in most of the cases and the guide strand gets loaded onto the RNA-induced silencing complex (RISC) to form the miRISC [36,37]. The RISC complex consists of Dicer, PACT, TRBP, Argonaute 2 protein (Ago2) and GW182 [36,38]. miRISC can now identify mRNA targets through base complementarity and can repress gene expression [39]. Repression of protein expression and mRNA decay requires the presence of the GW182 protein. GW182, along with the CCR4:NOT complex (CAF1 and NOT1), is required for promoting mRNA decay by miRISC [40]. Discovery of specific cytoplasmic foci, referred to as processing bodies (P-bodies), has brought in a new possible mechanism of mRNA decay [41]. Identifying the presence of untranslated mRNA in these P-bodies has led to various studies bridging the link between miRNA functions and these P-bodies. It has been elucidated that RISC recruits mRNA to P-bodies and promotes mRNA decay [42,43].

## 3. Functions of miRNAs

Even though miRNAs are coded only by about 3% of human genes, they can regulate about 30% of human protein coding genes [18] After discovering the role of the first identified miRNAs lin-4 and let-7 in cell cycle regulation in *C. elegans*, miRNA studies started focusing on the functional roles of miRNAs [16]. Since one miRNA can have multiple targets, they can regulate so many key pathways in humans. As a result, they can regulate various biological functions including metabolism, growth, development, immunity, etc. With their wide range of targets, it is of no wonder that they can regulate the pathogenesis of various diseases including cancer [44,45,46,47,48,49].

A review by Klein et al. has provided a detailed explanation regarding the role of miRNA in regulating gene expression during development in different species [50]. The involvement of miRNAs in controlling long-range guidance was first reported by Pinter and Hindges [51]. Studies completed in a mice retinal ganglion cell model that lack Dicer enzymes show that the depletion of miRNAs led to impaired cue expression or altered sensitivity of growth cones [52]. In 2011, Shibata et al. reported that miR-9 can regulate neogenesis in mouse telencephalon [53]. Along with miR-9, lin-4 has also been reported to regulate long-range guidance of the axonal projection [4]. miRNAs have been shown to regulate fasciculation and axon targeting [52]. Several defasciculated axon tracts were observed in zebrafish mutants lacking Dicer enzymes, whereas impaired axon targeting was observed in cells lacking miR-124 [54,55,56]. Bellon et al. has reported that miR-182 is involved in Slit2-mediated axon targeting [56]. It has also been reported that miRNAs are actively involved during cardiac development. The differentiation, proliferation and survival of all the different types of cells, cardiomyocytes, neural crest cells, epicardial cells and endocardial cells, from where the mammalian embryonic heart is derived from, are regulated by several miRNAs [57,58,59,60,61,62].

The role of miRNA in germline stem cell division in *Drosophila melanogaster* has been studied, showing that Dicer, the key player in miRNA biogenesis, is very important for the G1 to S phase transition of stem cells. The mutation in the Dicer1 gene reduced the germline cyst production dramatically. Scientists have also identified differential expression of miRNA during stem cell differentiation. The expression of the miR-290–295 cluster and miR-296 increases, whereas miR-21 and miR-22 expression decreases during stem cell differentiation [63]. An in vivo study using mice identified miR-7a2 as a key player in pituitary stem cell development. A loss-of-function study performed by deleting miR-7a2 in mice resulted in pituitary dysplasia, confirming the involvement of miRNA [64].

The function of miRNA in regulating the immune system has also been extensively researched. It has been reported that miRNAs can dysregulate the expression of certain key genes involved in prime immune functions, resulting in severe pathological consequences including autoimmunity diseases and cancers [65]. The first report on the role of miRNA in myeloid cell development was completed by Jonathan et al., where they reported that miR-223 regulates the granulocyte differentiation and activation and progenitor proliferation negatively. Upon mutation in the gene that transcribes miR-223, the number of granulocyte progenitors increased, and the mice developed distinct granulocyte compartments. In addition to this, miR-223 also regulates myeloid progenitor proliferation by targeting Mef2c, the transcription factor that promotes progenitor expansion [66]. miRNAs such as miR-155 and miR-146 are found to be overexpressed in response to certain inflammatory signals. A study by Ryan et al. confirms the active role of miR-155 in inflammation. miR-155 was seen to be overexpressed in macrophages upon exposure to inflammatory agents such as polyriboinosinic:polyribocytidylic acid or cytokine IFN-*β.* The AP-1 complex activated by the JNK pathway induced the transcriptional activation of the miR-155 coding gene [48]. In vivo studies show that miR-155 regulates the function of B cells, T cells and dendritic cells as well as the development of T helper cells [67]. miR-155 has also been identified as an oncogene, which may help us to find the link between inflammation and cancer [68,69]. A year later, the same group discovered that this miRNA links inflammation and certain pathologies associated with acute myeloid leukemia (AML). miR-155 was found to be upregulated in the bone marrow sample from patients with AML, and certain genes such as *Bach1, Sla, Cebpb*, etc., that are involved in hematopoietic development were targeted and repressed [70,71,72,73]. Studies also show that mice deficient in bic/miRNA-155 are immunodeficient, revealing its importance in regulating T helper cell differentiation and in optimal antibody responses [74,75]. IL-1-receptor-associated kinase 1 and TNF-receptor-associated factor 6 are the direct targets of miR-146 and are directly involved in inflammatory responses [76]. miR-150 is another miRNA that regulates the immune system by targeting c-Myb, a transcription factor that controls lymphocyte development. An in vivo study showed that miR-150 is expressed during the development in a stage-specific manner and binds to c-Myb at two conserved sequences in the 3′UTR, regulating gene expression [77]. It has been reported that miR-181a accentuates the sensitivity of T cells to antigens by modulating several genes in the T cell receptor signaling pathway [78].

Other targets for miRNAs are the genes involved in pathways related to primary and secondary pain. Fibromyalgia, which is associated with a lowered pain threshold, has been reported to have dysregulation in miRNA expression [79]. Compared to the control, patients with fibromyalgia have upregulation of miR-125a-5p and downregulation of miR-103a-3p, miR-107 and miR-130a-3p [80]. In migraine patients, levels of miR-126-3p, miR-155-5p and let-7g-5p were reported to be high compared to the control group [81].

One area where the role of miRNA is widely studied is in the initiation, development and progression of cancer. Since miRNAs are able to regulate the immune system, angiogenesis, inflammation, cell proliferation, cell growth, apoptosis, DNA repair, etc., it was of no wonder to scientists when they identified miRNA as the key player in tumorigenesis and tumor progression [82]. The dysregulation of miRNAs and how they control oncogenes as well as tumor-suppressing genes are discussed in detail in the following sections.

## 4. miRNA Deregulation in Cancer

The role of miRNAs in cancer is one of the emerging areas of study owing to its relevance in the prognosis, pathogenesis, diagnosis and treatment of cancer. Dysregulation of miRNA in cancer was first reported two decades ago by Calin et al. in chronic lymphocytic leukemia (CLL). A majority of B-cell CLL is associated with the deletion at chromosome 13q14, but scientists had failed to find any tumor-suppressing genes in that location that may be owing to CLL. Deletion and expression analysis performed by Calin et al. revealed that genes coding miR-15 and miR-16 are located within 30 kb loss in CLL, and these miRNAs are deleted or downregulated in most of the CLL cases, which shows the possible role of these miRNAs in CLL [83]. However, the mechanism of action was not clear until Cimmino et al. reported that B-cell lymphoma 2 protein, which is an antiapoptotic protein that is overexpressed in CLL, is the gene target for miR-15 and miR-16. Down regulation of miRNA expression increased the level of Bcl2 protein, negatively regulating the apoptotic effect of the miRNAs [46].

**Figure 1:**
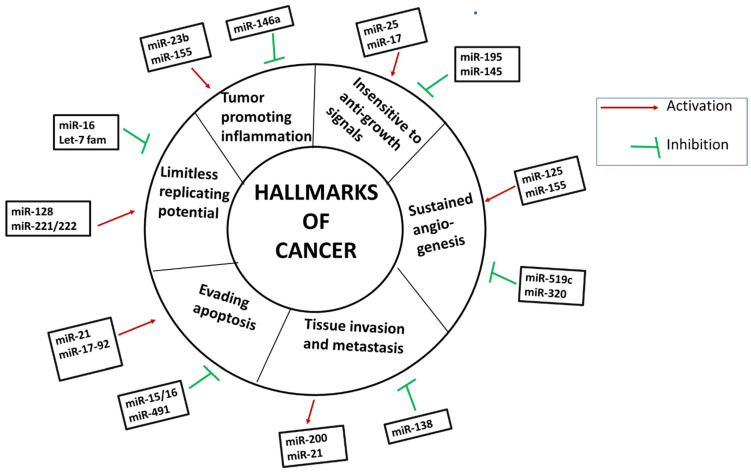
Different hallmarks of cancer being induced or inhibited by various miRNAs.

Later, it was reported that miRNAs are capable of regulating all the hallmarks of cancer. Therefore, as a tumor suppressor or as an oncomiR, various miRNAs can regulate tumorigenesis. Various hallmarks of cancer being regulated by miRNA are shown in Figure 1.

Dysregulation of miRNA in cancer can be due to epigenetic changes or alterations in miRNA biogenesis or polymorphism or mutation in the genes that code these miRNAs or chromosomal abnormalities. These can also happen conjointly, modulating the expression of miRNAs [84].

Defects in miRNA pathways/biogenesis have been documented in various cancers. Whole-exome sequencing of Wilms’ tumor, the most common childhood kidney cancer, identified missense mutations in genes that code Drosha and Dicer1. This reduced the expression of several tumor suppressor miRNAs including the whole let-7 family, identified as the mechanism of tumorigenesis in Wilms’ tumor [85].

Epigenetic modifications refer to the methylation of cytosine bases in DNA, posttranslational modifications of histone proteins and positioning of nucleosomes on the DNA. One of the very common epigenetic modifications observed in cancer is the hypermethylation of DNA as reported by [86,87,88]. miR-124 is a tumor suppressor miRNA that regulates cell proliferation, metastasis and invasion in colorectal cancer [89]. miRNA expression profiling and genetic studies using colorectal cancer cells lacking DNA methyltransferase enzymes show that the hypermethylation of CpG island leads to transcriptional inactivation of miR-124 [90]. Bandres et al. has also reported the hypermethylation of the promoter and hence the downregulation of miR-9-1, miR-129-2 and miR-137 in colorectal cancer [91]. This supports the link between the hypermethylation and modulation of miRNA expression in colorectal cancer. The hypermethylation of miRNA genes has also been reported in gastric cancer and renal cell carcinoma. The hypermethylation of genes encoding for miR-9 is reported in renal cell carcinoma compared to the healthy cells. This results in a nearly 30 month decrease in recurrence-free survival time, supporting the role of epigenetic modifications in metastatic recurrence [87]. Aberrant hypermethylation of miR-9 was also reported by Tsai et al. but in gastric cancer. Inactivation of miR-9 in gastric cancer induced cell proliferation, migration and invasion, confirming its role in gastric cancer progression [88].

Polymorphism of genes has been reported in various cancers [92]. Functional polymorphisms at the 3′ UTR of genes associated with several pathologies is assumed to be interfering with miRNA regulation. This could lead to an overexpression or downregulation of the genes downstream in the pathway. In addition to this, miRNA polymorphism can also lead to drug resistance by affecting miRNA-mediated regulation of a drug target gene [93].

RNA editing of a miRNA precursor can cause base changes in pri-miRNA or pre-miRNA sequences before it is exported to the cytoplasm. This is another post transcriptional modification that happens to miRNA in cancer followed by the modulation of its target gene [94].

### 4.1. miRNAs as Oncogenes

Genomic profiling studies reveal dysregulation of several miRNAs in cancer. miRNAs that are overexpressed, target tumor-suppressing genes and stimulate cell proliferation, angiogenesis, metastasis, etc., are referred to as oncomiRs [95].

In this section, we will discuss various oncomiRs that are overexpressed in different cancers and the genes that they target to establish their effect.

The miR-17-92 cluster, which generates six mature miRNAs (miR-17, miR-18a, miR-19a, miR-20a, miR-19b and miR-92a) and is located in intron 3 of the C13orf25 gene at 13q31.3, was observed to be aberrantly upregulated with occasional gene amplification in small cell lung cancer [96]. miR-92a, a component of the cluster, is reported to be upregulated in colon cancer and delivers anti-apoptotic activity by targeting Bcl-2 expression [97]. Another study completed by Zhu et al. shows that the miR-17-92 cluster is highly overexpressed in hepatocellular carcinoma tissues compared to non-tumorous liver tissue. Forced overexpression in vivo demonstrated enhanced cell proliferation, colony formation and invasiveness, supporting the oncogenic role of the miR-17-92 cluster [98]. The oncogenic effect of this cluster has also been studied in thyroid and renal cell carcinoma. miR-17 was found to be overexpressed in thyroid cancer, the inhibition of which caused complete growth arrest [99]. An in vitro study completed by Chow et al. discovered the overexpression of miR-17 and miR-20a in renal cell carcinoma [99].

miR-21 is another widely reported oncomiR that is overexpressed in most cancers. Upregulated levels of miR-21 have been reported in colorectal, gastric, prostate, breast, ovarian and pancreatic cancer [100,101,102,103,104,105,106]. In colorectal and gastric cancer, miR-21 plays a significant role in tumor initiation and progression, and its elevated level could be used as a prognostic marker [101,102,103]. Overexpression of miR-21 in breast cancer is associated with rapid cell growth and increased proliferation. Inhibiting the activity of miR-21 reduced the level of the anti-apoptotic protein Bcl2, resulting in increased apoptosis and decreased proliferation [104]. Downregulating miR-21 expression in pancreatic cancer inhibited cell proliferation and cell death by apoptosis and reversed drug resistance in ovarian cancer [105,106].

The loss of ZNF652 has been reported as one mechanism of breast cancer cell invasion and metastasis [107]. Genomic profiling has now shown that ZNF652 is a gene target of miR-155, another oncogenic miRNA, upregulated in breast cancer. Upregulated levels of miR-155 post-transcriptionally regulate ZNF652 and reduce its expression, which has be significantly correlated with increased local invasion [108]. Contrary to this, a recent study has reported the upregulation of ZN652 in HER2+ breast cancer cells [109]. This could be possibly due to the existence of other kinds of regulatory mechanisms in the gene.

Upregulated levels of miR-181 are reported in oral squamous cell carcinoma, breast cancer, prostate cancer and gastric cancer. Serving as an oncogene, miR-181 is associated with poor survival, vascular invasion and lymph node metastasis in oral squamous cell carcinoma [110]. In breast cancer, miR-181 downregulates the expression of PHLPP2 and INPP4B, which elevates the phosphorylation of Akt, resulting in Akt hyperactivation and promoting S-phase entry and cell proliferation [111]. Another study by Tian et al. elucidates another mechanism of action of miR-181 in breast cancer. Protein sprouty homolog 4 (SPRY4) has been identified as another target for miR-181, the downregulation of which is associated with the progression and poor prognosis of breast cancer [112]. In prostate cancer cells, miR-181 targets DAX-1 and induces cell proliferation [113]. These findings confirm the role of miR-181 as an oncomiR in tumorigenesis.

The miR-221/222 cluster has also been studied for its oncogenic role in cancer. Overexpression of miR-221/222 has been reported in liver tumorigenesis, where the CDK inhibitor p27 is targeted, inducing cancer cell growth, in breast cancer, where it regulates tumor development and progression by altering signaling pathways, and as a diagnostic and prognostic biomarker in colon and pancreatic cancer [100,114,115,116].

### 4.2. miRNAs as Tumor Suppressors

The first study on the dysregulation of miRNA in cancer completed by Calin et al. reported down regulation of miR-15 and miR-16 in CLL. A comparative study using normal samples showed an allelic loss at chromosome 13q14, where the two miR genes are located. Reduced expression of these miRNA is associated with the pathology of CLL, confirming its tumor-suppressing role [83]. Later, the tumor-suppressing mechanism of these miRNAs in CLL was identified via the Bcl2 gene, which codes for the antiapoptotic protein B [46]. A similar mode of regulation by targeting Bcl2 was also observed in human gastric and pancreatic cancer cells [117,118]. The downregulation/tumor-suppressing role of these miRNAs have later been reported in several other cancers. Fgf-2 and its receptor Fgfr1, which are associated with cancer cell survival, proliferation and migration, are post-transcriptionally regulated by miR-15 and miR-16 in prostate cancer. However, reduced expression of the miRNAs resulted in tumor expansion and invasiveness [119].

Let-7, the first identified miRNA, is a tumor suppressor, targeting key oncogenes such as Ras and Myc [45]. The tumor-suppressing role of let-7 family members has been implicated in various cancers such as lung, breast, gastric, colon, prostate, etc. Expression of let-7 is downregulated in non-small-cell lung cancer. Ectopic expression studies performed in mice induce cell cycle arrest and the cell death of lung cancer cells via Ras regulation. Hence, the role of let-7 in reducing the tumor burden in lung cancer was validated [120]. Lee and Dutta have reported HMGA2, another oncogene, as a potential target of let-7 in lung cancer [121]. This clearly indicates that one miRNA is capable of targeting multiple genes and can have a cumulative effect on the pathology of the disease. Buechner et al. studied the tumor-suppressing role of let-7 in neuroblastoma. Let-7 inhibited proliferation and clonogenic growth by targeting MYCN, a proto-oncogene [122]. Breast cancer cells also express low levels of let-7. A study performed on ER-positive breast tumor tissues show that let-7 acts as a tumor suppressor by inhibiting ERɑ-mediated cellular malignant growth. The inhibition of ERɑ by overexpressing let-7 can modulate the ER signaling pathway and can be used for breast cancer treatment [123].

With a potential to regulate more than 4000 gene products, the miR-29 family has been reported to be involved in various types of cancers related to almost all organizational systems: the respiratory system (lung cancer, lung adenocarcinoma, non-small cell lung cancer), nervous system (neuroblastoma and glioblastoma), digestive system (HCC, colon cancer, stomach cancer, esophageal cancer), muscular and skeletal system (osteoblastoma), reproductive system (ovarian cancer) and genitourinary system (bladder and prostate cancer). Downregulation of miR-29 has been associated with tumor progression and invasion in all these tumors, owing to its tumor-suppressing ability [124]. Drug resistance is a major hurdle in cancer treatment. It has been reported that the downregulation of miR-29 has been related to cisplatin resistance in ovarian cancer, the overexpression of which sensitizes the cells to the cisplatin treatment [125]. Lowered expression of miR-29 in osteosarcoma is associated with elevated cell proliferation and inhibited cell apoptosis. STAT3, upregulated in osteosarcoma, has been identified as the gene target for miR-29 [126]. Another study on miR-29 and osteosarcoma reports that miR-29 induces cell apoptosis by downregulating Bcl-2 and Mcl-1 and inducing E2F1 and E2F3 expression [127].

miR-34 is another tumor-suppressing miRNA, whose expression is down regulated in various cancers. Restoring the level of miR-34 in pancreatic cancer downregulated the expression of Bcl-2 and Notch 1/2 and inhibited cell growth and invasion [44]. Reduced expression of miR-34 has shown to regulate not only the pathogenesis but also the progression of epithelial ovarian cancer to the most advanced stages [128]. Forced expression of miR-34 in p53-mutant gastric cancer cells inhibited cell growth and developed apoptosis and chemosensitization, possibly by restoring p53′s function [129]. Both miR-101 and miR-122 are reported to be downregulated in hepatocellular carcinoma. miR-101 induced apoptosis in HepG2 cells, and miR-122 inhibited cell proliferation and migration by targeting Akt3 [130,131].

**Figure 2:**
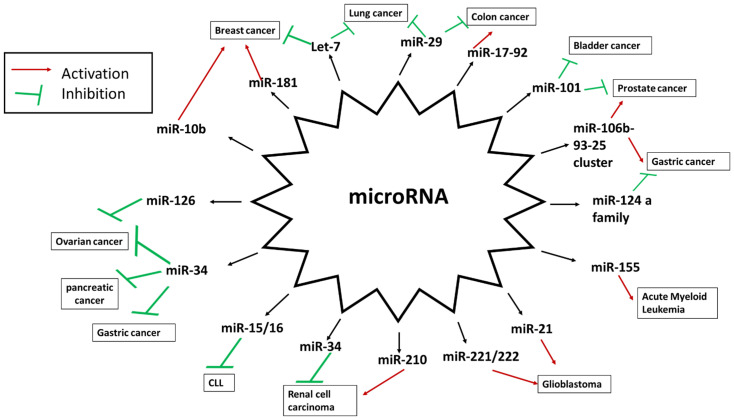
Different miRNAs regulating various cancers. One type of cancer is being regulated by mutliple miRNAs. A single miRNA can act as a tumor suppressor or oncomiR based on the cell type.

miRNAs that are downregulated and act as tumor suppressors and those that are upregulated and act as an oncomiR have been summarized in Figure 2.

The duplicity of miRNAs is commonly reported these days. The function of miRNA is cell-specific. miRNAs which function as tumor suppressors in one cancer type may act as an oncogene in another cancer cell [95]. miR-29 is one such miRNA, with two faces in cancer. Most studies report miR-29 as a tumor suppressor. However, an elevated expression of miR-29 has been reported in breast, AML-bearing cytoplasmic mutated nucleophosmin and colorectal cancer, where it has an upregulated level of expression [132,133,134].

## 5. miRNA Therapeutics

Out of the numerous treatments available for cancer, none of them gives complete eradication of the cancer cells from the patient. The side effects of the current treatment techniques are also questionable. The advanced understanding of the molecular mechanisms of cancer has helped us to target the cells at its root. Since miRNAs regulate many key players in tumorigenesis, tumor therapy using miRNAs is gaining widespread attention. The latest approach in miRNA therapeutics is mainly based on two strategies [135].

1. The inhibition of oncogenic miRNAs, and hence the restoration of the expression of tumor-suppressing genes that they target; or
2. Restoring the expression of tumor-suppressing miRNAs and hence inhibiting the oncogenes that they target

Various tools used for these strategies are explained in detail below.

### 5.1. Therapeutic Approach with Oncogenic miRNAs

Oncogenic miRNAs are usually upregulated in tumors and need to be suppressed to their normal level, so that its target genes, mostly the tumor suppressors, can be active and inhibit tumorigenesis or its progression. miRNAs that are synthesized to target the endogenous miRNAs are referred to as “anti-miRs” and are usually single-stranded oligonucleotides that have complementarity with the endogenous miRNA sequence. Therapeutic strategies against oncogenic miRNAs include: (i) anti-miRNA oligonucleotides, (ii) miRNA sponges, (iii) small molecule inhibitors, (iv) miRNA masking and (v) locked nucleic acid (LNA).

#### 5.1.1. Anti-miRNA Oligonucleotides (AMOs)

Anti-miR oligonucleotides are single-stranded chemically modified oligonucleotides that are 17–22 nt long. They are designed to be complementary to the target miRNA and hence bind to the mature miRNA, and thus prevent its interaction with the target gene. This will lead to the expression of the target gene, mostly a tumor suppressor. Once the AMO-miRNA duplex is formed, it will be cleaved by RNase-H [135]. A study performed in *C. elegans* with modified AMOs (2′O-methyl) complementary to let-7 miRNA induced let-7 loss of function [136].

#### 5.1.2. Modified AMOs

AMOs are often modified chemically to improve their stability. One such example of a modified AMO is Locked Nucleic Acid (LNA). By introducing an extra methylene bridge connecting the 2′-O atom and the 4′-C atom, the ribose moiety of the RNA is “locked” in C3′-endo or C2′-endo conformation, hence the name [137]. The locked conformation improves base stacking and increases thermal stability upon complementary binding with the single-stranded RNA target. The modified structure also provides good solubility and metabolic stability in vivo [138]. The efficacy of LNAs in suppressing oncomiRs has been studied in glioblastoma. miR-21, an anti-apoptotic factor highly upregulated in glioblastoma, induces tumor growth and progression [104,139]. In an in vitro study, the expression of miR-21 was completely silenced using LNA-anti-miR-21. Knockdown of miR-21 resulted in increased apoptosis of glioblastoma cells as well as sensitization to radiation-induced cell death [140,141]. This supports the effectiveness of LNA in miRNA therapeutics.

Another modified AMO that was introduced recently were the Antisense Phosphorodiamidate Morpholino Oligomers (PMO). PMOs are single-stranded DNA analogs consisting of a backbone of morpholine rings linked by phoshorodiamidate units. They function by binding to the target mRNA by complementarity pairing and thus exert transcriptional regulation. Recently, two PMO-based drugs, Eteplisren (Exondys 51) and Golodirsen (Vyondis 53 ^TM^), were approved by FDS for the treatment of Duchenne muscular dystrophy (DMD), owing to their safety and efficacy [142,143]. Later in 2021, Jayanta et al. reported that cell-penetrating PMO induced translational repression of genes involved in prostate and breast cancer [144].

Peptide Nucleic acids (PNA) are another group of modified oligos that can be used for anti-sense cancer therapies. PNA-mediated targeting of miRNAs is a novel cancer therapeutic approach due its high stability and superior hybridization affinity with DNA/RNA [145]. Lee et al. developed a photodynamic therapy based on a miRNA-responsive drug activation system. PNA with sequence complementarity to miR-21 was synthesized by conjugating it with a photosensitizer chlorin e6 (Ce6). Elevated levels of miR-21 in cancer cells facilitate the system to target the cancer cells, the binding to which releases the photosensitizer from the system, followed by the destruction of cancer cells by near-infrared radiation [146]. The PNS is a promising cancer therapeutic approach for targeting cancer cells based on the expression level of miRNAs.

Co-targeting two oncomiRs (miR-21 and miR-155) in lymphoma cell lines using PNAs and another anti-miR oligonucleotide called phosphorothioates (PS) has shown significant knockdown of the target miRNAs and reduced cell viability in lymphoma cells [147]. By substituting one non-bridging oxygen of the oligonucleotide with a hydrophobic sulfur atom, phosphorothioate, increases the stability as well as the availability of the nucleotide, thus making it a promising tool for therapeutics [148,149].

#### 5.1.3. miRNA Sponges

miRNA sponges, also called miRNA decoys, are competitive inhibitors with multiple binding sites for an endogenous miRNA and are used to prevent the interaction between the miRNA and its target mRNA. The ability of miRNA sponges to repress target miRNA is as strong as AMOs or LNAs. miRNA sponges can be designed with complementary heptameric seeds, such that a single sponge can target an entire miRNA seed family [150]. A study completed by Ma et al. used miRNA sponges to inhibit miR-9 expression in breast cancer cells, which effectively inhibited metastasis formation by upregulating CDH1 [151].

#### 5.1.4. Small Molecule Inhibitor

The crucial need to develop miRNA-based inhibition therapeutics led to the development of small molecules that can either inhibit miRNA biogenesis or inhibit miRNA–target interactions. By cellular screening, Gumireddy et al. came up with a small molecule inhibitor, azobenzene, acting not directly on the target miRNA, but on its biogenesis [152]. Recently, Melo et al. came up with a new molecule, enoxacin, an antibacterial compound that induces the production of tumor-suppressing miRNAs by binding to the miRNA biosynthesis protein TAR RNA-binding protein 2 (TRBP) [153]. Another study completed by Watashi et al. identified two compounds that modulate the miRNA pathway, polylysine (PLL) and trypaflavine (TPF). The mechanisms of action of these two compounds are different. PLL modulates miRNA activity by impairing Dicer activity, whereas TPF blocks small RNA loading into an Argonaute 2(Ago2) complex [154]. A very recent study by Hei et al. reported the synthesis of Fluoroquinolone derivatives that act as miR-21 inhibitors [155]. The stability and efficacy of these small molecule inhibitors should also be evaluated.

#### 5.1.5. miRNA Masking

In 2005, Xiao et al. developed a new approach called miRNA masking, which is a sequence with perfect complementarity to the miRNA binding site on the mRNA. miR mask forms a duplex with target mRNA with great affinity, making the binding site unavailable for the miRNA to bind to. They developed the miR mask based on the miR-1 and miR-133 target sites in the 3′UTR of HCN2 and HCN4. Masking the binding site on the 3′UTR enhanced the expression of HCN2/HCN4, confirming that this approach can be a potential miRNA therapeutic strategy [156].

### 5.2. Therapeutic Approach with Suppressor miRNAs

Tumor-suppressing miRNAs are always downregulated in cancer, which leads to the overexpression of their target oncogenes. Ectopic expression of tumor-suppressing miRNAs can be used to replace the lost miRNA level, aiming to inhibit cellular pathways that support oncogenesis. miRNA mimics are an effective tool that can be used to restore the function of tumor-suppressing miRNAs. miRNA mimics are small, chemically modified (2′-O’methoxy) double-stranded RNA molecules that mimic the endogenous mature miRNA molecules [84]. Several in vitro and in vivo modes have been used to study the efficiency of miRNA replacement therapy. The first study of its kind was competed by Aurora et al. using an orthotopic mouse lung cancer model, wherein they showed that the intranasal administration of let-7 reduced tumor formation in vivo, demonstrating let-7 as a potential miRNA-based therapeutic for lung cancer treatment [157]. miRNA replacement therapy was also successfully completed with miR-34 in non-small-cell lung cancer to reduce the tumor burden [158]. Commercially available miRNA mimics include pre-miR^TM^ miRNA precursors (Ambion) and miRIDIAN^TM^ microRNA mimics (Thermoscientific Dharmacon).

**Figure 3:**
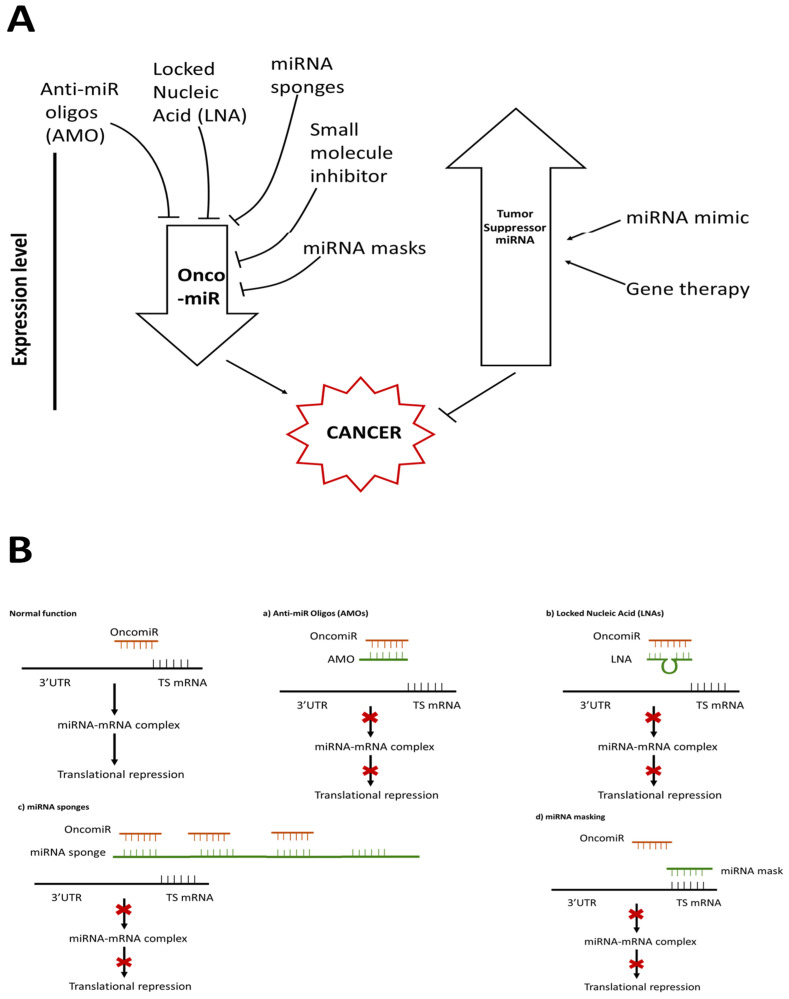
miRNA-based therapeutic strategies against cancer. (**A**). Several strategies are employed to repress the expression of oncomiRs and to promote the activity of tumor suppressor miRNAs. (**B**). miRNA-based therapeutic strategies and their mechanism of action against cancer. The effect of oncomiRs on tumor-suppressing mRNA (TS mRNA) can be controlled by (**a**) anti-miR oligos, (**b**) Locked Nucleic Acid (LNA), (**c**) miRNA sponges and (**d**) miRNA masking.

Figure 3 summarizes miRNA-based therapeutic approaches Figure 3A and their mechanisms of action Figure 3B.

## 6. miRNA Delivery Systems

The main hurdle to the clinical implementation of miRNA therapeutics is the lack of a foolproof miRNA delivery system. Even though miRNAs are small fragments of nucleotides, passive diffusion of these through the lipid membrane is not possible. In addition to this, their limited biological stability makes their delivery an obstacle to overcome. A delivery system that offers maximum miRNA availability with minimal toxicity would be an ideal choice.

The currently available systems for the efficient delivery of miRNA include non-viral vectors and viral vectors. The non-viral miRNA delivery system uses organic, inorganic or polymer-based carriers, whereas a viral-based delivery system usually uses lentiviruses, retroviruses, adenoviruses and adeno-associated viruses [159,160].

**Figure 4:**
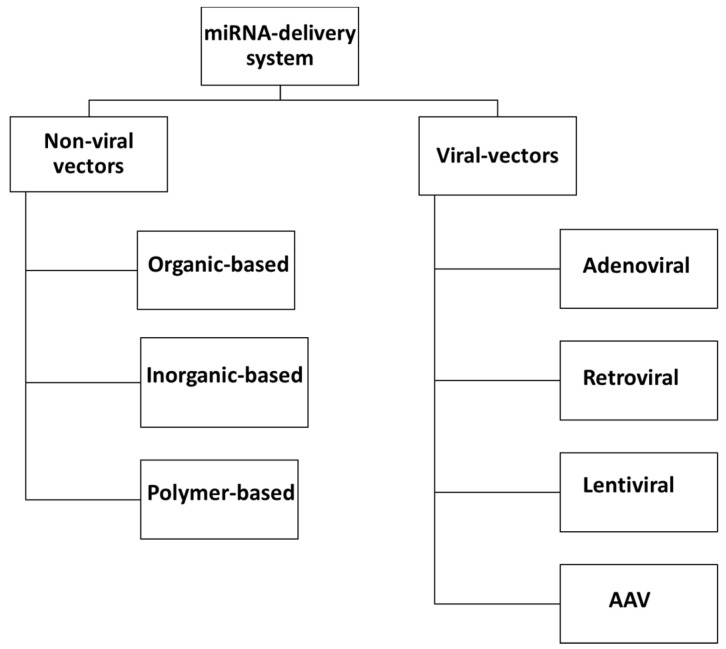
Different systems available for miRNA delivery can be categorized into non-viral vectors and viral vectors. The non-viral miRNA delivery system uses organic [161,162], inorganic [163] or polymer-based carriers [164,165], whereas viral-based delivery system uses lentiviruses [166,167,168], retroviruses [169], adenoviruses or adeno-associated viruses (AAV) [170].

Different systems available for miRNA delivery are summarized in the flowchart (Figure 4).

### 6.1. Non-Viral miRNA Delivery System

An efficient non-viral delivery system should be the one that transports endogenous miRNA or miRNA-expressing vectors by preventing nuclease-mediated degradation. The delivery can be facilitated either physically using techniques such as electroporation, a gene gun or an ultrasound or chemically using organic-based, inorganic-based or polymer-based carriers. Non-viral systems are usually less toxic and immunogenic, but with low transfection efficiency [160].

Organic-based carriers use lipid/nucleic acid complexes called liposomes that consist of encapsulated nucleic acids with an outer membrane-like surface. Cationic, anionic and neutral liposomes are available based on their charges, among which cationic liposomes are the most widely used, owing to their high affinity with the cell membrane [160]. It was reported that lipid-based drug delivery systems effectively exhibited therapeutic effects in both preclinical and clinical trials focused on cancer research [171]. Wu et al. have developed an efficient cationic liposome to restore the level of tumor-suppressing miRNA miR-29 in non-small-cell lung cancer. The systemic delivery of the liposome–miRNA complex was able to restore miR-29 levels in the cancer cell and inhibit tumor growth [161]. Dehghankelishadi et al. (2022) demonstrated the use of a biocompatible, high-density lipoprotein nanoparticle (HDL NPs) system to deliver radio-sensitizing RNA known as miR-34a. miR-34a-HDL NPs were conveniently developed using a fast microfluidics method. In vitro studies based on the administration of the miR-34a-HDL NPs to a head and neck cancer cell line showed that there was reduced metabolic activity, increased apoptotic activity and disruptions in the cell cycle. This provided evidence for the lipoprotein nanoparticles as a suitable delivery system for the treatment of head and neck cancer [172]. Sometimes, anionic and neutral liposomes are also used to overcome the instability caused by the interaction of cationic liposomes with serum proteins [162,168].

Inorganic materials that can be used for miRNA delivery include gold nanoparticles (AuNPs), Fe_3_O_4_-based nanoparticles and silica-based nanoparticles [165,173,174]. Gold nanoparticles are the most commonly used ones and have been employed to deliver miR-29b in myeloid cell leukemia and miR-130b in multiple myeloma cells [175,176].

Polymer-based delivery systems use naturally derived polymer-like cell-penetrating peptide (CPP) or synthetic polymers such as polyethylenimine (PEI), poly-lactide-co-glycolide (PLGA) and polyamidoamine (PAMAMs) dendrimers. Even though CPPs have minimal toxicity, they are easily degraded in the serum. Successful miRNA delivery (miR-29b) has been achieved using CPP in osteogenic stem cells [164]. Both high-molecular-weight and low-molecular-weight PEIs are used in delivery systems. However, small PEIs show less toxicity and damage to cell membranes and have been used to deliver miR-145 and miR-33a in colon cancer [173]. PLGA is an FDA-approved biodegradable polymer. PLGA nanoparticles modified with polyplexed PEI coating were used to deliver miR-26a in HepG2 cells [165]. PAMAMSs are positively charged polymers with high transfection efficiency [160]. PAMAMs have been used to deliver anti-miR-21 in glioblastoma cells to inhibit miR-21 activity [174].

**Figure 5:**
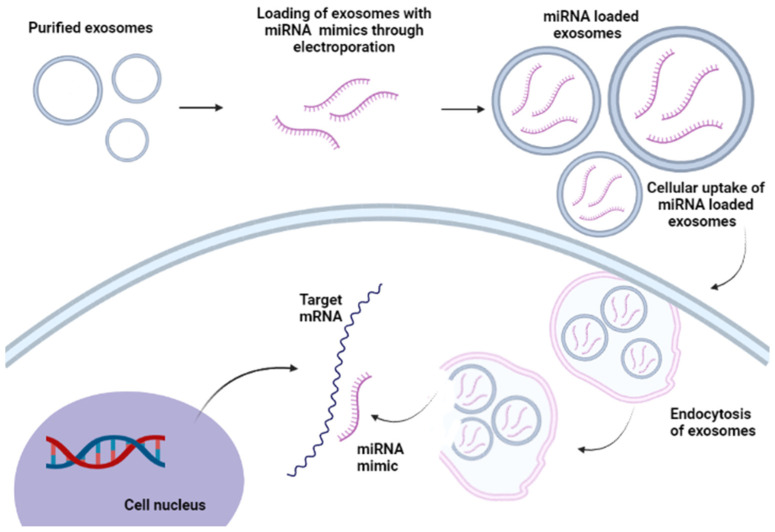
The cellular uptake of miRNA-loaded exosomes. Purified exosomes were transfected with the miRNA mimic to form miRNA-loaded exosomes which may enter cells through fusion or endocytosis, where they bind to target mRNA in the cytosol.

Another promising delivery system to transport miRNA is the use of outer membrane vesicles (OMVs) of *Escherichia coli* as nanoscale spherical vesicles for the treatment of cancer. Cui et al. (2022) demonstrated a cost-effective method to develop OMVs with over-expressed pre-miRNA for large-scale production. It was observed that OMVs containing over-expressed tRNALys-pre-miRNA could be used for tumor suppression. In vitro studies using eukaryotic cells demonstrated that pre-miRNA incorporated inside OMVs could be delivered and processed to mature miRNAs using tRNA scaffolds (Figure 5). In vivo characterization further showed the successful inhibition of the expression of target oncogenic CXCR4 using OMVtRNA-pre-miR-126 to suppress the growth of breast cancer cells [177]. The use of exosomes as delivery carriers for miRNA may be effective in overcoming in vivo miRNA degradation since exosomes can efficiently cross the biological carrier and sustain communication with target cells. The biogenesis of exosomes and targeting mechanisms showed that the exosomes could optimize the expression of specific endogenous miRNAs and promote the regulation of several physiological mechanisms including cancer cell apoptosis [178].

### 6.2. Viral-Based miRNA Delivery System

Genetically modified viruses can be utilized to deliver desired oligonucleotides to elevate the expression of miRNA or to deliver anti-miR to suppress target miRNA expression [179]. Genetic modification makes the virus unable to replicate and hence more safe. Viral-based miRNA delivery systems use lentiviruses, retroviruses, adenoviruses and adeno-associated viruses [160].

Retroviral vectors are formed from retroviruses from the Retroviridae family. Retroviral vectors are commonly isolated from Moloney murine leukemia viruses (MoMLVs), consisting of a simple genome expressing Gag, Pol and Env proteins flanked by long terminal repeats (LTR) [180]. Retroviruses are RNA viruses with 7–11 KB genome size. Genetic sequences up to 8 KB genetic can be carried by the virus and are incorporated into the host cell during the mitotic phase of the cell cycle. Hence, this delivery system can be used only for dividing cells. Once incorporated into the host genome, stable as well as increased expression of the exogenous sequence is observed. Retroviral vectors have been successfully used to induce the expression of miR-138 in mouse embryonic fibroblasts [169].

Lentivirus is a subgroup of retrovirus. Lentiviral vectors can also transfer 8 KB genetic sequences to the host cell. Lentiviruses differ from retroviruses due to their ability to infect both dividing and non-dividing cells. This feature makes lentiviruses capable of infecting differentiated cells to treat neurological disorders [179]. Lee et al. were successfully able to transfect miR-145 into corneal epithelial progenitor cells using lentiviral vectors [47]. Systemic in vivo lentiviral delivery of miR-15a/16 was found to reduce malignancy in a mouse model of chronic lymphocytic leukemia [166]. A prominent example of the use of the viral vector delivery platform to deliver miRNA is the lentiviral vector platform known as pSLIK (single lentivector for inducible knockdown), which was developed by Shin et al. (2016) [167]. This system allowed the tetracycline-dependent regulated expression of microRNA-like short hairpin RNAs. The delivery of these microRNA-like structures using the lentiviral platform led to the repression of the expression of the heterotrimeric G proteins Gα12 and Gα13. Moreover, the addition of a GFP transgene to the microRNA-like structure allowed the reconstitution of heterologous mRNA in targeted gene knockdown experiments. Such studies suggested that viral vectors might effectively be used to deliver miRNA to both enhance or inhibit mRNA expressions [167].

Adenoviruses are double-stranded DNA viruses and can transfer large genes of up to 38 KB. However, they cannot incorporate the exogenous genes into the host genome [160]. Adeno-associated viruses (AAVs) are single-stranded DNA viruses which can transfer only up to 4.8 KB gene sequences. However, this is hardly an issue given the small size of miRNA genes. Moreover, AAVs can infect both dividing and non-dividing cells, which broadens their applications [170].

Desirable features of miRNA delivery vehicles are having a high loading capacity, good stability, enhanced half-life in circulation, slow degradation of the miRNA cargo and minimal toxicity. Viral vectors face challenges such as immunogenicity and cytotoxicity despite having high transfection efficiency. In contrast, non-viral delivery systems face low transfection efficiencies, but this could be overcome by chemical modifications such as positively charged synthetic polyamidoamine (PAMAM) dendrimers, which showed higher transfection efficiency and lower cytotoxicity when compared to other polymers. Similarly, the half-life of lipid nanoparticles could be increased by conjugation with polyethylene glycol (PEG) [181].

## 7. Drug Intervention on miRNA Therapy in Cancer

miRNAs can exhibit therapeutic potential either alone or in combination with other additional treatment strategies. Combination therapy of miRNA with other antitumor therapeutic modalities such as chemotherapy drugs has been documented to target a broader range of tumors, induce therapeutic efficacy and overcome drug resistance. Several studies showed synergistic effects of treatments and significantly increased the efficacy of anti-tumor therapy when used concurrently compared to either a single application of miRNA or a chemotherapy drug. Chemotherapy drugs may slow the growth and spread of cancer cells. However, over time, the cells develop resistance to the drugs via several distinct mechanisms and pathways.

For example, the sensitivity to chemotherapeutic drugs was known to be modulated by miR-21. Furthermore, it has been shown that miR-21 is an oncogenic miRNA in several types of cancers. It lowers the expression of PTEN, thus enhancing AKT-mediated activation of Bcl-2 signaling and causing chemoresistance in cancer cells. Significant anticancer efficacy was demonstrated by the co-delivery of the miR-21 inhibitor conjugated to doxorubicin encapsulated in star-branched copolymers made up of polylactic acid (PLA) and polydimethylaminoethyl methacrylate (PDMAEMA). Co-treatment of LN299 glioma cells with the miR-21 inhibitor and doxorubicin was reported to reduce the tumor volume by nine times when compared to treatment with either miR-21 or doxorubicin treatment alone, indicating that this is a potential therapeutic strategy [182]. Earlier research on miR-29b and bortezomib, an anticancer drug used to treat multiple myeloma, showed that miR-29b with the SP1 transcription factor could induce the susceptibility of the cells to bortezomib and induce apoptosis through the PI3K-AKT signaling pathway. A combination treatment of miR-29b and bortezomib produced significant pro-apoptotic effects when compared to the control group. In addition, Wang et al. (2011) discovered that the application of miR-21 in combination with bortezomib, doxorubicin and dexamethasone had synergistic effects in eradicating multiple myeloma cells and was shown to be more effective when compared to the application of chemotherapeutic drugs alone [183].

A novel anticancer drug, Paclitaxel (PTX), was used to treat several types of cancers. Shi et al. (2014) constructed a co-delivery system combining the PTX drug with miR-34a and examined the synergistic effects. The combination of miR-34a and PTX loaded in functional solid lipid nanoparticles (miSLNs-34a/PTX) enhanced the antitumor activity, significantly reduced the tumor growth and eliminated the cancer cell population in the tumor-bearing murine model [184]. In a recent study by Gandham et al. (2022), a therapeutic combination of Let7b with PTX significantly increased antitumor activity in a multidrug-resistant model of epithelial ovarian cancer (EOC). Additionally, further evidence showed that combinatorial therapy was safe for repeated administration. This cutting-edge strategy of cellular reprogramming of tumor cells employing therapeutically relevant miRNA mimics in combination with the chemotherapy drug could result in more beneficial therapeutic outcomes for patients with advanced-stage relapsed EOC [185].

Normann et al. (2021) reported that miRNA could inhibit breast cancer cells in vitro when combined with HER2-targeting drugs. A high-throughput screening of 1626 miRNA mimics and inhibitors in combination with trastuzumab and lapatinib was conducted in HER2+ breast cancer cells to determine whether miRNAs could render HER2+ cells more sensitive to the treatment. The tumor-suppressing activity of miR-101-5P was supported by the finding that higher expression of this miRNA predicted a better outcome in HER2+ breast cancer patients. The results uncovered that miRNAs could make HER2+ breast cancer cells more susceptible to targeted therapy and have the potential to improve existing therapies for HER2+ breast cancer by combining targeted drugs with miRNAs [186].

Docetaxel is a distinct broad-spectrum chemotherapeutic agent. It is used to treat various cancer types, particularly solid tumors [187,188]. miRNA-34a has been characterized as a potent tumor suppressor [189]. Zhang et al. (2017) designed a core-shell nanocarrier coated with cationic albumin to deliver miRNA-34a and docetaxel into breast cancer cells concurrently to increase treatment efficacy. The co-delivery of both compounds was found to be more effective at treating metastatic breast cancer compared to miRNA-34a or docetaxel alone, where it reduced the expression of Bcl-2, the anti-apoptosis gene, effectively prevented tumor cell migration, and promoted cell apoptosis and cytotoxicity [190].

## 8. Patent Updates for miRNA in Cancer Therapy

Various miRNAs appear to be promising therapeutic targets from a scientific perspective [191]. As seen by the sharp rise in the number of patent applications over the past 10 years, it is perhaps not surprising that the growth in the scientific study of miRNA biology has spurred a large number of innovations in this field. The use of miRNA as a therapeutic entity is generating a lot of attention. The involvement of miRNAs in cancer is an area where intellectual property (IP) is expanding daily. Pharmaceutical companies are making huge investments in research on miRNAs and their potential clinical applications. Other than the developed countries, pharmaceutical companies are looking forward to the market of emerging industrial countries as their market is growing quicker. As a result, it might be challenging for both developed and emerging countries to benefit from IP. Research suggests that miRNAs are critical regulators of gene activation in the development and spread of cancer. Currently, scientists are employing miRNA expression profiles to categorize cancers and define miRNA markers as diagnostics to establish a good prognosis. miRNA research has become a very prominent area as a result of their biological and medical significance, and the number of patent applications pertaining to miRNAs is constantly increasing.

Based on the patent search using the query “microRNA cancer”, there were 21,549 patents resulting from the search. A list of selected patents utilizing miRNA as potential therapeutics against cancers granted in the US, European and other countries is presented in Table 1.

## 9. miRNA-Based Clinical Trials for Cancer Therapy

The first miRNA that is rapidly moving from the bench to the clinic is LNA-antagomiR-122, also known as Miravirsen, for the treatment of hepatitis C virus infections [192]. It is a modified oligonucleotide made up of 15 nucleotides that binds to and inhibits miR-122. Miravirsen has now completed the phase II clinical trial (NCT01200420), which assessed its safety and effectiveness in patients. To date, there are only 10 miRNAs that have progressed to clinical trials, and none of them has been entered into the clinicaltrials.gov database for phase III trials. Four biopharmaceutical companies such as miRNA Therapeutics Inc, MiRagen Therapeutics, EnGeneIC and Regulus therapeutic are primarily responsible for the development of miRNA-based therapies for cancer (Table 2).

The first-in-class miRNA therapy for cancer is MRX34, manufactured by miRNA Therapeutics Inc. MRX34 is a double-stranded miR-34 mimic encapsulated in a liposome-formulated nanoparticle [193]. In most cancer cells, miRNA-34a is normally downregulated and functions as a tumor suppressor [194]. It influences more than 20 oncogenes involved in apoptosis and cell cycle arrest. It has been shown that high levels of miRNA-34a in cancers would reduce the proliferative capacity of the cells, which indicates a potential therapeutic approach for the treatment of cancers [195]. MRX34 demonstrated potent efficacies in a wide range of cancers such as melanoma, renal cell carcinoma, multiple myeloma and non-small-cell lung cancer (NSCLC) (NCT01829971). Since antisense oligodeoxynucleotides could not diffuse into cells, a liposomal nanoparticle was used to deliver the miRNA-34a for the therapy of cancers. In a multi-center phase I study, MRX34 was administered intravenously for five consecutive days, followed by two weeks off for three cycles [196]. Unfortunately, this trial was terminated in 2016 due to immune-related severe adverse events which resulted in four deaths [193]. Nevertheless, the development of MRX34 provided a practical strategy for finding miRNA drugs by enhancing the permeability of antisense oligonucleotides into the cells with cutting-edge formulations such as liposomal nanoparticles. This clinical trial offers guidelines for the use of MRX34 in oncotherapy and more importantly serves as a proof-of-concept for miRNA-based cancer therapy. Most patients who received MRX34 treatment with dexamethasone premedication experienced a tolerable toxicity profile, [193,196], highlighting the need to be aware of potential adverse reactions from this class of drugs, particularly immune-mediated events that might not always be visible in pre-clinical models. To fortify this, Zhang et al. (2021) have recently discussed the delayed development of miRNA from a pharmacological perspective, contending that the reaction to the onset of adverse events, and subsequently the dysregulated miRNA therapeutic clinical trial pipeline, is a result of “too many targets for miRNA effect” [197].

In 2014, EnGeneIC Limited generated an alternative carrier based on bacteria-derived nanocells. This non-living bacterial minicell technology has been used by Reid et al. (2016) to create TargomiR, a miRNA replacement therapy in thoracic cancer patients [198]. This led to a MesomiR-1 (miR-16 mimic) phase I study of TargomiR as second- or third-line treatment in patients with malignant pleural mesothelioma and NSCLC (NCT02369198). miR-16 family members have been identified as acting as tumor suppressors, with downregulation being observed in a wide range of cancers. The restoration of miR-16 expression by miRNA mimics has been shown to inhibit tumor cell proliferation [199]. In a phase I open-label dose-escalation study, TargomiRs treatment was shown to be tolerable when patients were administered intravenously with five billion nanocells loaded with miR-16 mimics once a week [200]. Results from the trial were promising, with no adverse effects being observed, which then allowed for the continuation of the phase II clinical trial.

miR-155 was abundantly expressed in lymphoma cells, and it played a vital role in enhancing the growth and survival of cancer cells [201]. Thus, the suppression of miR-155 is necessary to return the cells to resume their normal function. In 2016, MiRagen Therapeutics conducted a phase I clinical trial of Cobomarsen, an inhibitor of miR-155, and investigated its safety, tolerability and pharmacokinetics in patients with cutaneous T cell lymphoma (CTCL), mycosis fungoides (MF), chronic lymphocytic leukemia (CLL), diffuse large B cell lymphoma (DLBCL) and adult T cell leukemia/lymphoma (ATLL) (NCT02580552) [202]. Preliminary results showed that intratumoral injections of Cobomarsen for a period of up to 15 days could ameliorate the cutaneous lesions without causing any obvious side effects [203]. As a result, a phase II clinical trial was continued in 2018 to examine the effectiveness and safety of Cobomarsen for the treatment of CTCL and MF (NCT03713320). Although the trial was halted in December 2020 due to business concerns, it also encouraged scientists to develop miRNA for cancer therapy.

Another miRNA, miR-10b, plays an essential role in the invasion and metastasis of several cancers [204]. Over 100 studies on miR-10b have revealed its central role in various metastatic tumors. Therefore, suppressing its activity could be a therapeutic strategy. miR-10b was one of the most expressed miRNAs in metastatic breast cancers, and it was highly linked with metastasis [205]. The miR-10b inhibitor was shown to significantly inhibit breast cancer metastasis when administered in vivo [206]. A miR-10b is also a promising target for glioblastoma therapy, involved in regulating cell migration, invasion and metastasis [207]. The clinical significance of miR-10b is its role in metastatic tumors, where antagomiR-10b reduced metastasis in tumor-bearing mice by restoring Hoxd10 gene expression [208]. In high-grade gliomas, miR-10b was expressed at higher levels in glioblastomas than in normal brain tissues [207]. This study tested the hypothesis that in primary glioma, miR-10b expression patterns could serve as a prognostic and diagnostic marker. The phenotypic and genotypic heterogeneity of various glioma subtypes were also described in this study. Guessous et al. (2013) confirmed that miR-10b was upregulated in human glioblastoma tissues, glioblastoma cells and stem cell lines, while the inhibition of miR-10b would reduce cancer cell proliferation and inhibit invasion and migration [207]. Hence, targeting miR-10b might be a good strategy for glioblastoma treatment. Furthermore, based on the critical function of anti-miR-10b in the inhibition of glioblastoma growth, a clinical trial was undertaken for evaluating the level of expression of microRNA-10b in patients with gliomas (NCT01849952). More studies are needed to confirm the effects of anti-glioblastoma in the clinic.

Additionally, the sensitivity of individual primary tumors to anti-miR-10b treatment in vitro could be examined given the important role anti-miR-10b plays in suppressing established glioblastoma growth. TransCode Therapeutics Inc. developed the anti-miR-10b nanoparticle formulation (TTX-MC138) for the treatment of breast cancer, and it is now undergoing evaluation in the safety and dose escalation phase. On the other hand, Regulus in 2019 announced their new miRNA drug candidate, RGLS5579, a novel oligonucleotide designed to target miR-10b for potential trials in patients diagnosed with glioblastoma multiforme, the most aggressive form of brain cancer, with a median survival of approximately 14.6 months [209].

## 10. Future Prospects and Challenges

To date, researchers have not developed any cancer therapy that can wipe off cancer cells from the system. With advancements in research, we are now able to elucidate the mechanisms of cancer initiation and progression at the molecular level. This has shed light on the tight link between miRNAs and cancer, and miRNA therapeutics can be the future of cancer therapy. The main hurdle that is faced in miRNA-based therapy is target specificity. As one miRNA can target many genes, there will be off-target effects which need to be addressed. It has been reported that off-target gene silencing can lead to neuro and immunotoxicity and may reduce its therapeutic effects [210]. Intensive studies will be necessary to elucidate the multiple gene targets of miRNAs and to study the off-target effects. A study completed in *D. melanogaster* emphasizes the off-target issues of RNAi techniques and suggests to use co-inhibition or co-induction techniques [211]. Targeting a gene with a single miRNA would not be sufficient to bring out the desired effect. Co-inhibition or co-induction using a combination of miRNAs could be a possible solution for this, and it may be helpful to provide a cumulative effect. In general, a deep understanding of the interaction between the miRNA and the target genome and the signaling pathways that are modulated would be beneficial in experimental designs.

Another challenge would be to choose the perfect cost-effective miRNA delivery system, with high efficacy and low toxicity. The delivery system should be optimized to deliver the exogenous miRNA and for it to exert its effect by integrating with the genome.

miRNA-based therapy could also be used in conjunction with already established treatments such as chemotherapy or radiation. The ability of certain miRNAs in chemo sensitization would be beneficial in combination therapies. Several in vitro studies and a few in vivo studies have already shown the effectiveness of miRNA therapeutics in reversing certain hallmarks of cancer. Several pre-clinical and clinical studies are ongoing, and in the future, we could expect to develop miRNA sequences based on an individual genome to cater personalized cancer therapy.

## 11. Conclusions

Recent studies clearly indicate the possibility of regulating miRNAs to modulate oncogenes and tumor-suppressing genes. In vivo studies have also established its potential to inhibit tumor growth. miRNA profiling has shown that miRNA levels in cancer cells vary from their healthy counterparts. The level of tumor-suppressing miRNAs and oncomiRs is downregulated and upregulated respectively in cancer cells compared to the normal healthy ones. By suppressing the effect of oncomiRs and by inducing the expression of tumor-suppressing miRNAs, tumorigenesis and tumor progression can be controlled to an extent. Viral or non-viral vectors can be used for the delivery of miRNA into the system. Major issues of the current therapy are the side effects of chemodrugs as well as the drug resistance that cancer cells developed due to its long exposure. Combination therapy using miRNA and chemo drugs can be utilized to have an effective way to treat cancer cells. By resolving the challenges of target specificity and miRNA delivery, we can drive miRNA translational research from the bench to the clinic much faster.

## Figures and Tables

**Table 1:** Patents issued for miRNA in cancer therapy.

Patent No.	Country of Origin	Patent Statement	Inventors	Applicant	Publication Date
**WO2022169922A1**	USA	Compositions and methods for treating disease associated with dux4 overexpression. miR-675 inhibits DUX4 expression	Saad Nizar, Harper Scott	Res Inst Nationwide Childrens Hospital, USA	11 August 2022
**WO2022170133A1**	USA	MicroRNA liver cancer markers and uses thereof	Rotroff Daniel, Aucejo Federico	Cleveland Clinic, USA	11 August 2022
**WO2022164028A1**	Korea	Pharmaceutical composition, for preventing or treating liver fibrosis, containing mir-486-5p as an active ingredient	Jung Youngmi et al.	Pusan National University	4 August 2022
**WO2022153846A1**	Japan	Composition for treating cancer or suppressing angiogenesis, including a microRNA agonist selected from the group consisting of miR-139-3p agonists, miR-214-3p agonists or a combination thereof.	Iwamoto Hideki et al.	Kurume University	21 July 2022
**WO2022136226A1**	France	miRNA composition comprising 11 specific miRNAs and its use in the treatment of cancer	Jauliac Sébastien	Institut National de la Santé et de la Recherche Médicale	30 June 2022
**WO2022103026A1**	Korea	Biomarker for diagnosing metastasis of cervical cancer and uses thereof (hsa-miR-1228-5p, hsa-miR-3200-3p, hsa-miR-146a-3p, hsa-miR-33a-5p, hsa-miR-6815-5p)	Cho O Yeon	Ajou University	19 May 2022
**CN114432331A**	China	Application of miR-138mimic in preparation of ovarian cancer stem cell medicine for inhibiting YAP and WWTR1 high expression	Zhou Qi et al.	Chongqing University Cancer Hospital	6 May 2022
**WO2022072336A1**	USA	Drug-like molecules and methods for the therapeutic targeting of microrna-21	Shortridge Matthew	University of Washington	7 April 2022
**CN114177293A**	China	Application of targeting miR-493/HIF-1alpha/PDK1 in preparation of drug-resistant drugs	Liu Wenjing et al.	Henan Cancer Hospital	15 March 2022
**CN114099684A**	China	Application of miR-32-5p in preparation of medicine for improving the sensitivity of tumor cells to dihydroartemisinin	Li Yujie et al.	Institute of Materia Medica of CAMS	1 March 2022
**CN114053294A**	China	Application of miR-150 and exosomes loaded with miR-150 simulants for the preparation of medicine for treating colorectal cancer	Zhou Jian et al.	Zhongshan Hospital Fudan University	18 February 2022
**CN113755601A**	China	Melanoma molecular marker and application thereof in the early diagnosis and treatment of melanoma (miR-196a, miR-27a-5p and miR-27b-5p)	Kong Yun et al.	Beijing Baiaosike Biomedical Technology Co., Ltd. Kangtai Medical Laboratory Service Hebei Co., Ltd.	7 December 2021
**CN113713104A**	China	Application of miR-345-3p in preparation of breast cancer treatment drug	Chen Tingmei, Zeng Qian	International Institute of In Vitro Diagnostics Chongqing Medical University	30 November 2021
**CN113637762A**	China	Application of miRNAs related to melanoma in the diagnosis and treatment of melanoma (miR-1321 and miR-139-5p)	Cui Minglu et al.	Mld Biotech Co., Ltd.	12 November 2021
**CN113621707A**	China	Application of hsa-miR-190b for the preparation of products for diagnosing and/or treating tumors	Yin Mengxiong, Shang Chuangeng, Ma Jiahui, Song Shuliang; Zhang E	University Shandong	9 November 2021
**N113637762A**	China	Application of miRNAs related to melanoma in the diagnosis and treatment of melanoma	Cui Minglu et al.	Beijing Baiaosike Biomedical Technology Co., Ltd.	15 October 2021
**CN113476618A**	China	Application of miR-199a-3p in the preparation of medicine for treating nasopharyngeal carcinoma	Luo Haiqing et al.	Guangdong Medical University	8 October 2021
**CN112941183A**	China	Application of non-coding gene miR-187-5p in primary liver cancer diagnosis and treatment	Zhou Jun et al.	Wuhan University of Science and Technology	11 June 2021
**CN112791187A**	China	Application of miR-142-5p in the preparation of medicine for treating chronic myelogenous leukemia	Wang Shuzhen et al.	China Pharmaceutical University	14 May 2021
**CN111961727A**	China	Application of miR-588 and the target gene thereof in gastric cancer	Xu Songxiao et al.	Zhejiang Cancer Hospital	20 November 2020
**CN111961722A**	China	Application of miR-887 in breast cancer diagnosis and treatment	He Jingsong	Peking University Shenzhen Hospital China	20 November 2020
**WO2018181877 A1**	Japan	Cancer stem cell growth inhibitor using miRNA	Xin Wu et al.	Cancer Stem Tech Inc, Japan	4th October 2018
**WO2018157026A1**	USA	Treatment of tumors with miRNA targeting CDK4/CDK6	Amriti R Lulla and Wafik S EL-Deiry	Institute for Cancer Research and The Research Institute of Fox Chase Cancer Center, USA	30th August 2018
**CN108452307 A**	China	Application of human miRNA-493-3p inhibitor in preparing medicine for treating renal fibrosis	Rui Du et al.	The Fourth Military Medical University, China	28th August 2018
**US7642348 B2**	USA	miRNA for the diagnosis, prognosis and treatment of prostate cancer; linear amplification and labeling for hybridization techniques such as Luminex and microarray analysis	Itzhak Bentwich et al.	Rosetta Genomics	5 January 2010
**7825229 B2**	USA	miRNAs; diagnosis, prognosis, and treatments; drug screening; linear amplification and labeling for hybridization techniques such as Luminex and microarray analysis; gene expression inhibition	Itzhak Bentwich et al.	Rosetta Genomics	2 November 2010

**Table 2:** Clinical research progress of miRNA-related drugs.

Target miRNA	Drug Name	Company	Disease	Study	Clinical Trial Number, Phase Status
**miR-122**	Miravirsen	SantarisPharma	Hepatitis C virus (HCV) infection	Multiple Ascending Dose Study of Miravirsen in Treatment-Naïve Chronic Hepatitis C Subjects	NCT01200420; Phase II (Completed)
**miR-34**	MRX34	miRNA Therapeutics	Liver cancer, Lymphoma, melanoma	A multicentre phase I study of mrx34, microrna miR-rx34 liposomal injection. Five serious immune-related adverse events Pharmacodynamics study of MRX34, microRNA liposomal injection in melanoma patients with biopsy-accessible lesions (MRX34-102)	NCT01829971; Phase 1 (Terminated) NCT02862145; Phase 1 (Withdrawn)
**miR-16**	MesomiR-1	EnGeneIC	Mesothelioma, lung cancer	MesomiR 1: A Phase I study of TargomiRs as second- or third-line treatment for patients with recurrent MPM and NSCLC	NCT02369198; Phase 1 (Completed)
**miR-155**	Cobomarsen (MRG-106)	miRagen therapeutics	T-cell lymphoma/ mycosis fungoides	Safety, tolerability and pharmacokinetics of MRG-106 in patients with mycosis fungoides (MF), CLL, DLBCL or ATLL Efficacy and safety of Cobomarsen (MRG-106) vs. active comparator in subjects with mycosis fungoides (SOLAR)	NCT02580552; Phase 1 (Completed) NCT03713320; Phase 2 (Terminated)
**miR-10b**		Regulus Therapeutics	Glioma	Evaluating the expression levels of microRNA-10b in patients with gliomas	NCT01849952; (recruiting)
